# Radiomics of Multiparametric MRI to Predict Biochemical Recurrence of Localized Prostate Cancer After Radiation Therapy

**DOI:** 10.3389/fonc.2020.00731

**Published:** 2020-05-12

**Authors:** Qiu-Zi Zhong, Liu-Hua Long, An Liu, Chun-Mei Li, Xia Xiu, Xiu-Yu Hou, Qin-Hong Wu, Hong Gao, Yong-Gang Xu, Ting Zhao, Dan Wang, Hai-Lei Lin, Xiang-Yan Sha, Wei-Hu Wang, Min Chen, Gao-Feng Li

**Affiliations:** ^1^Department of Radiation Oncology, National Center of Gerontology, Beijing Hospital, Beijing, China; ^2^Key Laboratory of Carcinogenesis and Translational Research (Ministry of Education / Beijing), Department of Radiation Oncology, Peking University Cancer Hospital and Institute, Beijing, China; ^3^Department of Radiation Oncology, City of Hope Medical Center, Duarte, CA, United States; ^4^Department of Radiology, Beijing Hospital, National Center of Gerontology, Beijing, China

**Keywords:** imaging, prostate neoplasm, radiotherapy, radiomics, prognosis

## Abstract

**Background:** To identify multiparametric magnetic resonance imaging (mp-MRI)-based radiomics features as prognostic factors in patients with localized prostate cancer after radiotherapy.

**Methods:**From 2011 to 2016, a total of 91 consecutive patients with T1-4N0M0 prostate cancer were identified and divided into two cohorts for an adaptive boosting (Adaboost) model (training cohort: *n* = 73; test cohort: *n* = 18). All patients were treated with neoadjuvant endocrine therapy followed by radiotherapy. The optimal feature set, identified through an Inception-Resnet v2 network, consisted of a combination of T1, T2, and diffusion-weighted imaging (DWI) MR series. Through a Wilcoxon sign rank test, a total of 45 distinct signatures were extracted from 1,536 radiomics features and used in our Adaboost model.

**Results:**Among 91 patients, 29 (32%) were classified as biochemical recurrence (BCR) and 62 (68%) as non-BCR. Once trained, the model demonstrated a predictive classification accuracy of 50.0 and 86.1% respectively for BCR and non-BCR groups on our test samples. The overall classification accuracy of the test cohort was 74.1%. The highest classification accuracy was 77.8% between three-fold cross-validation. The areas under the curve (AUC) of receiver operating characteristic curve (ROC) indices for the training and test cohorts were 0.99 and 0.73, respectively.

**Conclusion:**The potential of multiparametric MRI-based radiomics to predict the BCR of localized prostate cancer patients was demonstrated in this manuscript. This analysis provided additional prognostic factors based on routine MR images and holds the potential to contribute to precision medicine and inform treatment management.

## Introduction

Prostate cancer is the second most common cancer in men worldwide. In recent years, there has been a rapid increase in the incidence of prostate cancer in China, with an average annual growth rate of 12.07% (1). A common treatment option for localized prostate cancer includes endocrine therapy combined with radiation therapy. Biochemical recurrence (BCR) occurs in a significant number of patients after radiotherapy, with the 5–8-years biochemical relapse-free survival rates being 65–85% in patients treated with intensity-modulated radiotherapy (IMRT) and image-guided radiation therapy (IGRT) (2). Pretreatment identification of BCR in patients with localized prostate cancer can be useful to predict prognosis and guide treatment decisions. Factors that have shown prognostic value in previous work include absolute baseline prostate-specific antigen (PSA), PSA doubling time (PSADT), tumor stage (T-stage), and pathologic findings (including Gleason score and lymph node status) (3).

Multiparametric magnetic resonance imaging (mp-MRI) has shown promising results in the diagnosis, localization, risk stratification, and staging of prostate cancer. It has also afforded opportunities for focal treatment for prostate cancer (4, 5). However, MRI is rarely used to predict the efficacy of radiotherapy, especially in BCR prediction settings. Ginsburg SB et al. analyzed sixteen patients and found computer–extracted texture features on T2-weight MRI are useful for predicting the likelihood of developing biochemical recurrence following radiation therapy (6). A recent study also demonstrated that T2-weight Haralick features appeared to be strongly associated with BCR for peripheral zone prostate cancer (7).

Radiomics has attracted increased attention in recent years. The concept was introduced <5 years ago but has been explored by many researchers in the clinical medicine and biomedical engineering communities globally. By converting medical images into high-dimensional, mineable data via high-throughput extraction of quantitative features, additional information beyond the original raw images can be obtained by subsequent data analyses (8–10). Although radiomics studies have been extensively performed in multiple cancer sites, such as lung and colorectal cancers, few have been conducted for prostate cancer. Among the studies in which radiomics was applied for prostate cancer, only a few involved the use of radiomics to predict the prognosis after radiotherapy (6, 7, 11–20). Therefore, the aim of this study was to investigate the correlation of mp-MRI features in predicting the prognosis of localized prostate cancer after radiotherapy.

## Patients and Methods

### Patients

This retrospective analysis was approved by an institutional review board, and the informed consent requirement was waived. The entire cohort of this study was retrieved from the records of the Institutional Picture Archiving and Communication System (NEUPACS version 5.5, Shenyang, Liaoning, China) between October 2011 and June 2016. Baseline clinical pathologic data, including age, histological grade, T-stage, N-stage, PSA, and dates of baseline MRI were obtained from medical records. Tumor staging was defined according to the American Joint Committee on Cancer (AJCC) TNM staging system manual, 7th edition. Gleason score and risk group were defined according to the National Comprehensive Cancer Network (NCCN) guidelines, version 2.2017.

### Inclusion Criteria

- Patients with histologically confirmed T1-4N0M0 prostate carcinoma without evidence of lymphatic or distant metastases at diagnosis.
- Patients who had undergone a pretreatment 3.0 Tesla MRI scan (Achieva TX, Philips Healthcare, Best, The Netherlands).
- Pretreatment MRI images (including CET1-w and T2-w images) were available in the PACS.
- Patients received neoadjuvant endocrine therapy for 1–3 months.
- Patients were followed up every 1–3 months during the first 2 years, every 6 months in years 2–5, and annually thereafter.
- Biochemical failures were diagnosed by Phoenix consensus: PSA increase by 2 ng/mL or more above the nadir PSA after EBRT with or without hormone therapy (21).
- Clinical data, such as age, histology and overall stage, were available.

### Exclusion Criteria

- Patients who received radiotherapy, chemotherapy, or endocrine therapy before their first MRI scan.
- Patient MRI was not acquired with a 3.0 T MR scanner.
- Incomplete clinical data.

Of the 384 consecutive patients treated for prostate cancer during this time period, a total of 91 patients (mean age, 73.8 ± 7.8 years; range, 50–90 years) met the inclusion criteria and were enrolled into the study. The radiation regimen included 74–80 Gy delivered in 37–40 fractions by using IMRT/IGRT techniques. Sixty-two patients (68%) were classified as non-BCR and 29 (32%) as BCR. The median follow-up time was 56.2 months (range, 10–131 months). The detailed clinical characteristics of the patients are listed in Table 1. There were no statistical differences between the BCR and non-BCR groups in terms of age, Gleason score or TNM stage. However, the initial PSA level, pre-radiotherapy PSA level, and NCCN risk groups differed significantly between the two cohorts (*p* < 0.05, see Table 1).

**Table 1:** Characteristics of patients and tumors.

**Characteristics**	**Non-BCR *N* = 66**	**BCR *N* = 27**	***P***
Age, mean ± SD, years	74.3 ± 6.9	72.4 ± 9.5	0.054
Initial PSA level, mean ± SD, ng/ml	36.1 ± 52.4	84.2 ± 73.2	0.032
Pre-radiotherapy PSA level, mean ± SD	2.7 ± 7.8	26.4 ± 76.3	0.000
Gleason score group			0.069
Group 1	14(21.2)	3(11.1)	
Group 2	18(27.3)	4(14.8)	
Group 3	9(13.6)	2(7.4)	
Group 4	12(18.2)	7(25.9)	
Group 5	13(19.7)	11(40.7)	
NCCN risk			0.010
Low	6(9.1)	0(0)	
Intermediate	20(30.3)	1(3.7)	
High	34(51.5)	20(74.1)	
Very high	6(9.1)	6(22.2)	
T stage			0.698
T1	2(3.0)	0(0)	
T2	44(66.7)	9(33.3)	
T3	17(25.8)	15(55.6)	
T4	3(4.5)	3(11.1)	

*Data show number of patients (%) unless otherwise indicated. BCR, biochemical recurrence; PSA, prostate-specific antigen; NCCN, National Comprehensive Cancer Network*.

The 91 patients were divided into training and test cohorts at a ratio of 4:1 by using computer-generated random numbers in order to construct and verify the Adaboost model. Seventy-three (80%) patients were allocated to the training cohort, and 18 (20%) were allocated to the test cohort.

### MRI Protocols

All patients underwent a pretreatment 3.0 T MRI scan. For feature selection, axial T2-weighted images, axial T1-weighted images, and axial diffusion-weighted images (DWI) archived in the PACS were used. All images were in raw format without any post-processing or normalization.

The acquisition parameters were as follows: axial T2-weighted spin-echo images (repetition time/echo time [TR/TE]: 1,599/100 ms, field of view [FOV] = 22 × 22 cm, number of excitations/average [NEX] = 1.5, slice thickness = 4 mm, spacing = 1.0 mm), axial T1-weighted spin-echo images (TR/TE: 480/10 ms, FOV = 25 × 32 cm, NEX = 1.5, slice thickness = 4 mm, spacing = 1.0 mm), and axial DWI SE-EPI images (TR/TE: 2,905/61, FOV = 24 × 24 cm, NEX = 3.0, slice thickness = 4 mm, spacing = 1.0 mm, b = 0, 1,000 s/mm^2^).

### Region of Interest

**Figure 1:**
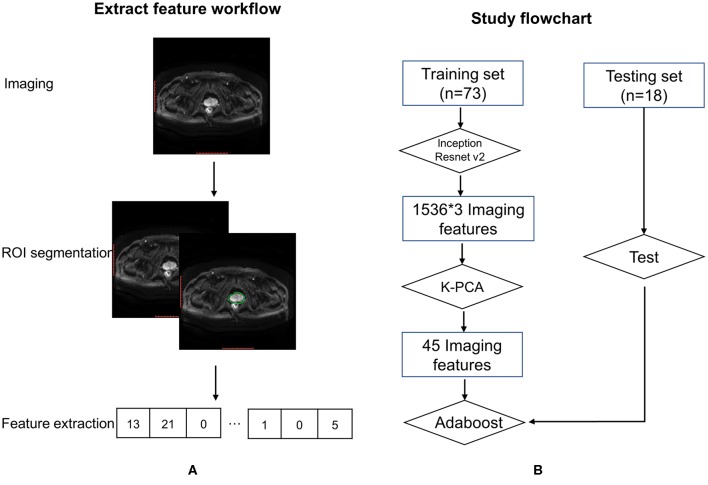
Feature extraction workflow **(A)** and study flowchart **(B)**.

For every patient in the training cohort, multiple MR images of prostate tumors were acquired in three scanning modes: T1, T2, and DWI. In the radiomics analysis, segmentation of region of interest (ROI) was one of the most critical steps to reduce uncertainty. The process was automated as much as possible with minimal manual intervention. Images were automatically registered via DICOM so the ROI could be copied from one image to another. To maximize consistency, the prostate ROI was contoured using T1 and T2 axial images by the same radiation oncologist and reviewed/approved by another senior radiation oncologist. Voxels outside the prostate contour were removed from all three MR image types to reduce computational complexity. Only voxels inside the prostate ROI were maintained for radiomics image analysis. The MRI ROI and an integral flow chart are as shown in Figure 1.

### Feature Extraction

Radiomics images were derived from content-based image properties, which could be categorized into three levels, i.e., pixel-level, object-level, and semantic-level, based on the number of image attributes captured by the features and the biological interpretability of the features (22). The semantic-level features were generated to predict the BCR in prostate images.

Semantic-level features were in a higher level of the information hierarchy, meaning these features capture interpretable concepts. A deep learning method allowed us to choose the most effective combination of the lower level features to distill information that was valuable for prediction. By using deep learning to elevate the lower level features to the semantic-level features, the accuracy of survival class predictions can be improved (23). With the biological variations present in the radiomics images extracted from large training data sets, the development of semantic-level descriptors became feasible by employing convolutional neural networks (CNNs). In addition, the inception-family ability to detect higher dimension features was maximized using the algorithm outlined by Szegedy et al. (24).

Our model used the Inception-Resnet-v2 network introduced by Szegedy et al. (25). The algorithm was a CNN that represented the state of the art in terms of accuracy in the ILSVRC image classification challenge. Inception-ResNet-v2 was a variation of the Inception V3 model that borrowed ideas from the Microsoft ResNets networks, optimizing speed of convergence while avoiding the exploding gradient problem. Residual connections included shortcuts in models that, as mentioned above, allowed researchers to train even deeper networks that achieved better performance. This also allowed a significant simplification of the inception blocks. The hyperparameters used in this study are listed in Table 2:

**Table 2:** Hyperparameters used in feature extraction.

**Momentum**	**Initial learning rate**	**Decay**	**Number of epochs per decay**	**Weight decay rate**	**End learning rate**	**Batch size**
0.9	0.01	0.95	2	0.00004	0.00001	32

### Feature Selection

The data consisted of 3 MRI sequences (DWI, T1, T2) for every patient. The missing data in (DWI, T1, T2) was set to 0. The biochemical recurrence prediction ability of each sequence was analyzed separately and jointly.

**Figure 2:**
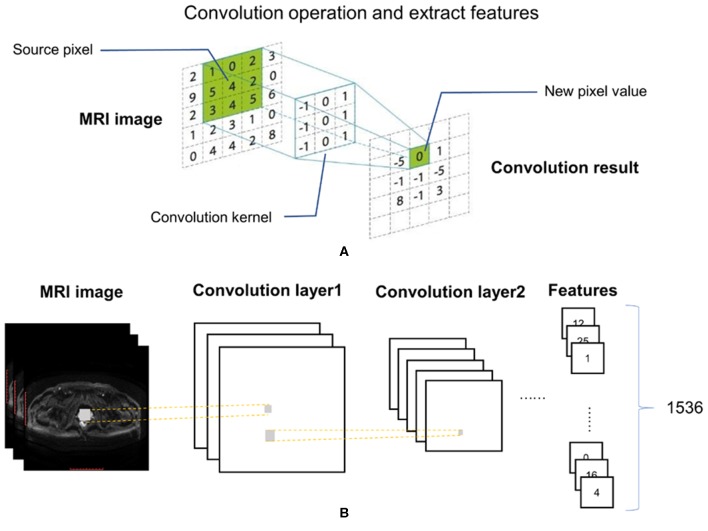
Convolutional operation **(A)** and feature extraction with Inception-Resnet V2 **(B)**.

An Inception-Resnet v2 network was used to analyze the MR images. The input of this network included each of the three MRI sequences and patient labels. The output was a total of 1,536 quantitative features. Feature kernels were convolved with the pixel matrices of each of the MRI sequences to obtain a series of smaller matrices, which were labeled as convolution operation (Figure 2A). Feature selection was applied with the Inception-Resnet v2 network (Figure 2B), to identify an optimal feature combination, collecting the correlation between each pixel and its neighborhood. In the following step, the mean values from each slice were adopted as input to the next machine-learning classifiers.

**Figure 3:**
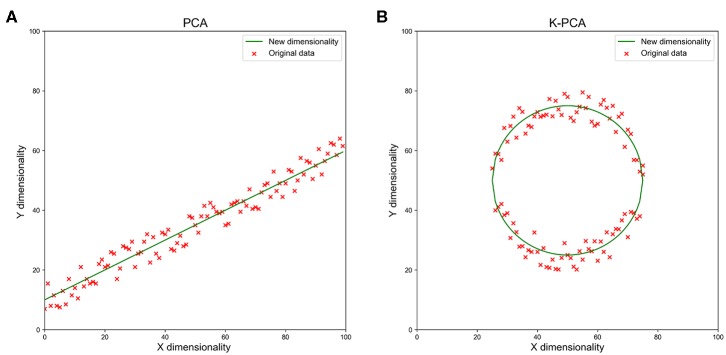
A simple example of reducing the feature dimensionality from 2d to 1d with PCA **(A)** and k-PCA **(B)**. We used the green line as a new dimension to replace the original x and y dimensions when the data showed the clumped distribution observed in the figures. With the increase in dimensions, the data loss reduced rapidly.

Finally, kernel principal component analysis (k-PCA) was performed to reduce the number of dimensions in the feature space. In the field of multivariate statistics, k-PCA is an extension of PCA using techniques of kernel methods. A simple example of k-PCA is as shown in Figure 3. Using a kernel, the originally linear operations of PCA were performed in a reproducing kernel Hilbert space, so that k-PCA could extract nonlinear information from the data.

### Statistical Analysis

Individual variables were analyzed for significant differences using the *t*-test and the Wilcoxon-Mann-Whitney test for non-normally distributed parameters. Group results were reported as mean standard deviations. Intergroup differences were compared with a paired difference test; P < 0.05 was considered statistically significant.

### Adaboost Model

An Adaboost model was constructed to conduct this classification task. The 45 radiomics features (15 features from each of the three sequences) were used as the input of the neural network for training. Since the 91 sample sets were randomly divided into training cohort (80%) and test cohort (20%), the samples in the training and test cohorts were considered to come from the same distribution.

Region of interest (ROI) segmentation, MRI normalization and feature extraction and selection were performed using AIMED version 1.7.5 (https://www.blothealth.com). Model construction was conducted using AIMED version 1.7.6 (https://www.blothealth.com).

## Results

**Figure 4:**
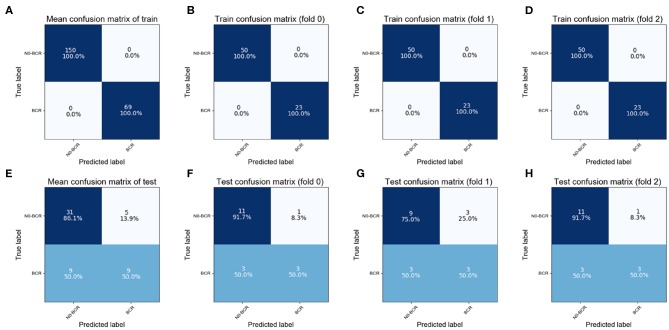
Confusion Matrix; **(A–D)** the training confusion matrix; **(E–H)** the test confusion matrix.

In the Adaboost model, the classification was performed via an iterative 3-fold cross-validation process, and the resulting mean and standard deviation values for the classification accuracy were computed. In addition, receiver operating characteristic (ROC) analysis and area under the curve (AUC) measurements were used to quantify the extracted features' ability to predict prostate cancer. All of the parameters in our model were obtained by cross-validation. A confusion matrix was used to describe the performance of a classification model as illustrated in Figure 4. The matrix was a table with two rows and two columns that reported the number of false positives, false negatives, true positives (TP), and true negatives (TN).

The training cohort contained 23 positive and 50 negative samples in each fold. The correct classification rate of training samples was 100% (Figures 4A–D), which was attributable to the ensemble learning model. In the test cohort, six positive and 12 negative samples participated in each fold. As demonstrated in the findings (the bottom of Figure 4), the overall classification accuracy of the test cohort was 74.1% (accuracy = (TP+TN)/total). The correct classification rate for positive samples was 50.0% and the correct classification rate for negative samples was 86.1%. The highest classification accuracy was 77.8% for 3-fold cross-validation.

**Figure 5:**
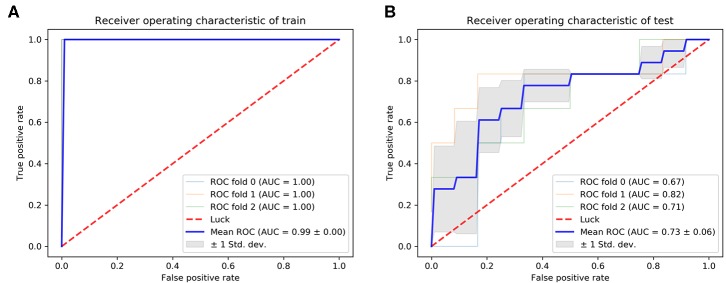
ROC curve; **(A)** the training ROC; **(B)** the test ROC.

Classifier output requires validation of data integrity. One figure of merit representing the neural network to sample data coherence is the receiver operating characteristic plot. It indicates how the false-positive and true-positive rates relate as thresholds of outputs. It has a value from 0 to 1, with 1 being that false positive and true positive are completely unrelated. The AUC of the ROC curve was used as the evaluation index for model quality. In our final model, the mean AUC of the training cohort and validation cohort was 0.99 (Figure 5A). The mean AUC of the test cohort was 0.73. The AUC of each fold was 0.67, 0.82, 0.71 (Figure 5B).

## Discussion

The mechanism underlying the association between radiomics features and cancer prognoses is not fully understood. However, it has been suggested that intratumor heterogeneity may be one of the major factors because of its important role in diagnosis and prognosis (26). This view is consistent with our understanding of cancer cells' micro-environmental distribution. Malignant tissues consist of heterogeneous cell populations with distinct molecular and micro-environmental characteristics, in contrast to non-malignant tissues. In traditional clinical staging, it is very difficult to take intratumor heterogeneity into consideration (9). However, a radiomics approach can consistently extract higher dimensional image features intrinsic to shape and texture, which are highly correlated with intratumor heterogeneity. Therefore, radiomics signatures should be explored in cancer prognosis in conjunction with other clinical staging variables (27).

Machine-learning techniques have previously been shown to be useful in predicting patient prognosis in several cancers (28–30). For instance, researchers have developed fully automated microscopic pathology image features to predict non-small cell lung cancer prognosis (29). Other groups of investigators have associated features in mp-MRI with the prognosis of rectal cancer (28). In this study, we demonstrated that the extracted image features from MRI can predict patient BCR. These quantitative image features are impossible to spot by manual inspection. However, computerized methods can efficiently and effectively identify such features. Since MR images are routinely used in current clinical practice, our classifiers could be easily implemented in routine practice.

In our study, an automated workflow to identify objective features from MR images was designed. A neural network-based machine-learning classifier was built and validated to predict the BCR of prostate cancer patients. The results showed significant promise for the use of mp-MRI as one of the tools to predict the prognosis of prostate cancer. Previously, the progression of BCR after radiotherapy for prostate cancer relied on clinical parameters, such as PSA, PSADT, TNM stage, and pathologic findings (including Gleason score, surgical margin status, and lymph node status). In recent years, radiomics has been extensively explored in terms of tumor diagnosis, treatment and prognosis. The most widely used imaging modality in radiomics research is CT, which can quantify tissue density (8). Unlike CT, MRI can detect tumor density and reveal the physiologic characteristics of tumors (31). In addition, MRI provides better tissue contrast, has multiplanar capacity, and exhibits fewer artifacts from radiation and bony autonomy. MRI allows the tumor volume to be delineated more accurately (32), especially for prostate cancer. It also affords a variety of scanning parameters and techniques. In order to reduce the associated uncertainty, we used a consistent MRI scan technique in our study. This was the first study demonstrating that the prognosis of localized prostate cancer after radiotherapy could be evaluated using a radiomics approach based on mp-MRI.

There are several limitations to this study. First, only MR images were included in the model. Other imaging techniques, such as the prostate-specific membrane antigen (PSMA) ligand (68) Ga-PET/CT (PSMA PET/CT), can potentially add additional clinical features into the model. Another limitation was the potential biased distribution of samples due to limited number of patients included in this study. Ideally, equal numbers of samples from BCR and non-BCR groups are recommended in the training set to train an unbiased classifier. The testing cohort could be unbalanced but the training set is not recommended to be unbalanced. However, given the distribution in our patient group, that was not feasible in this study and could potentially result in inaccuracy of the model parameters. This might contribute to the relatively inferior prediction performance of the positive sample group which certainly warrants further investigation and improvement with larger sample size. With the correct classification rate of 86.1% for the negative samples, we expect that the overall classification accurate will increase more with the improvement in the predication of positive sample group. Furthermore, the model parameters may not readily apply to images from other institutions with different scanners and settings. In other words, selection bias may have occurred since strict criteria were used and the randomization hypothesis was compromised, which could affect the model training. For the first attempt, we used strict inclusion criteria to select 91 patients with only clinical stage T1-4N0M0. Our model has not been tested against N1 and M1 patients. For future study, we plan to increase the patient sample size by relaxing our inclusion criteria and further improve the accuracy of the BCR group and potentially expand this study into a multicenter research project.

In conclusion, our study evaluated the relationship between pretreatment mp-MRI radiomic features and biochemical recurrence in patients with localized prostate cancer. Through a systematic analysis of mp-MRI-based radiomics, we were able to establish and validate a model with improved predictive value over conventional imaging metrics. Our model could facilitate prognostic predictions based on routine MRI, further contributing to precision oncology and enhanced quality of care.

## Data Availability Statement

The datasets used and analyzed during the current study are available from the corresponding author on a reasonable request.

## Ethics Statement

The studies involving human participants were reviewed and approved by Ethics Committee of Beijing hospital. The patients/participants provided their written informed consent to participate in this study. Written informed consent was obtained from the individual(s) for the publication of any potentially identifiable images or data included in this article.

## Author Contributions

Q-ZZ, L-HL, AL, and G-FL: conception and design. Q-ZZ, C-ML, XX, X-YH, Q-HW, HG, Y-GX, and TZ: collection and assembly of data. Q-ZZ, L-HL, DW, H-LL, X-YS, W-HW, and MC: data analysis and interpretation. All authors wrote the manuscript and final approval of manuscript.

## Conflict of Interest

The authors declare that the research was conducted in the absence of any commercial or financial relationships that could be construed as a potential conflict of interest.
